# Surgical complications after caesarean section: A population-based cohort study

**DOI:** 10.1371/journal.pone.0258222

**Published:** 2021-10-05

**Authors:** Charlotta Larsson, Elin Djuvfelt, Anna Lindam, Katarina Tunón, Pär Nordin

**Affiliations:** 1 Department of Surgical and Perioperative Sciences, Umeå University Hospital and Östersund Hospital, Östersund, Sweden; 2 Östersund Hospital, Östersund, Sweden; 3 Department of Public Health and Clinical Medicine, Unit of Research, Education and Development, Östersund Hospital, Umeå University, Umeå, Sweden; 4 Department of Clinical Science, Obstetrics and Gynaecology, Umeå University Hospital, Umeå, Sweden; Poissy-Saint Germain Hospital/Versailles Saint Quentin University, FRANCE

## Abstract

**Background:**

The rate of caesarean section without medical indication is rising but the risk for surgical complications has not been fully explored.

**Methods:**

Altogether 79 052 women from the Swedish Medical Birth Register who delivered by caesarean section only from 2005 through 2016 were identified and compared with a control group of women delivering vaginally only from the same register and the same period of time. By cross-linking data with the National Patient Register the risks for bowel obstruction, incisional hernia and abdominal pain were analysed, as well as risk factors for these complications. We also analysed acute complications, uterine rupture, and placenta praevia.

**Findings:**

Caesarean section is associated with an increased risk for bowel obstruction (OR 2.92; CI 2.55–3.34), surgery for bowel obstruction (OR 2.12; CI 1.70–2.65), incisional hernia (OR 2.71; CI 2.46–3.00), surgery for incisional hernia (OR 3.35; CI 2.68–4.18), and abdominal pain (OR 1.41; CI 1.38–1.44). Smoking, obesity, and more than one section delivery added significantly to the risk for these complications.

**Interpretation:**

Caesarean section is considered a safe procedure, but awareness of the risk for serious complications is important when deciding on mode of delivery. In this study, more than one section, obesity and smoking significantly increased the risk for complications after caesarean section. Prevention of smoking and obesity among fertile women worldwide must continue to be a high priority.

## Introduction

The caesarean section rate is rising rapidly and continuously in many parts of the world. In 1985, WHO stated that the ideal rate on a population level should be 10–15% with no decrease in maternal or perinatal mortality obtained with rates above that [1]. This was based on the evidence available at the time, and the validity of this statement has since been questioned and an updated version with a softer statement that caesarean section should be performed when needed, focusing more on the lack of evidence regarding optimal rates and how to improve this knowledge in the future [2,3]. The rate has since then increased to 24.5% in Western Europe, 32% in North America and 41% in South America [4,5]. Since the procedure is often performed on indications other than medical, a complete understanding of the risks of this abdominal surgical procedure is most important [4].

To date, no comprehensive study covering surgical complications after caesarean section has been published. The most common complication is massive bleeding, reported in 7% of cases [6]. Smaller studies report damage to the inner organs such as the urinary tract, bowel, and large vessels, in a small number of cases [7]. Abdominal pain after caesarean section appears to be a significant but varying problem; two systematic reviews reported rates from 4% to 42% [8,9]. Bowel obstruction was reported in 0.05 to 0.2% in two large studies, but it is unclear when in relation to the delivery obstruction occurred, and whether surgery was required [10]. Previous studies have shown an increase in incisional hernia repair rate after multiple caesareans sections [11]. The rare but severe complications uterine rupture and placenta praevia have increased in recent years, possibly due to the increase in caesarean section rate [12,13].

The aim of this study was to analyse the risk for surgical complications after caesarean section at a population level using nationwide registers. A control group of women delivering vaginally only was used for comparison.

## Methods

### Study design

This observational population-based study used data from two nationwide patient registers; the Swedish National Patient Register and The Swedish Medical Birth Register [14].

The Swedish National Patient Register was started 1964 and has had complete coverage of inpatients since 1987. Since 2001 it has also included outpatients, but primary care is still not included. There is approximately 1% missing data for inpatient care. During the study period, the register included data on all discharge diagnoses and surgical intervention codes according to the International Classification of Diseases (ICD) [15,16].

The Swedish Medical Birth Register includes prospectively collected data on all pregnancies, deliveries, and neonatal periods since 1973. Coverage is almost complete except for the variables BMI, smoking habit, and whether the delivery was elective or emergency [17].

All Swedish citizens have a unique personal identification number which makes it possible to cross-link two registers and follow individuals over time regardless of where in Sweden they live and seek medical care [18].

Ethics approval was granted by the Regional Ethics Committee at Umeå University, Sweden (Dnr 2015-410-31, Dnr 2016-12-32). The STROBE checklist for observational studies was followed [19].

### Participants

The study period was from 2005 through 2016. The main study group consisted of all women delivering by caesarean section only, and the control group consisted of all women delivering vaginally only. To ensure no mixing with previous deliveries, all women in the study had their first delivery during the study period. Each woman could only be registered in the study once, although she could have more than one diagnosis and more than one delivery. In the analyses of complications occurring within 42 days, all deliveries were counted separately.

Exclusion criteria were those having both vaginal and caesarean deliveries.

### Procedures

The primary outcome was risk for surgical complications after caesarean section compared to vaginal delivery.

The surgical complications studied were: bowel obstruction; incisional hernia; abdominal pain; and short-term complications occurring within 42 days after delivery (puerperium). Each diagnosis was only counted once for each woman *i*.*e*., the first time it occurred in the register. Women in the study group and the control group (extracted from the Swedish Medical Birth Register) were cross-linked with the ICD-10 diagnosis and intervention codes from the National Patient Register. (See S1 and S2 Tables for all diagnosis codes.) Since data were available, we also studied the risk for placenta praevia and uterine rupture. Time from delivery to first diagnosis/intervention for primiparas was also analysed.

A secondary outcome was risk factors for surgical complications after caesarean section. To identify risk factors, and for the adjusted comparison, the population was divided into the following subgroups: infant birthweight dichotomised into above or below median; maternal age divided into < 30 years, 30–34 years and ≥ 35 years; number of deliveries per woman divided into one delivery or more than one delivery; and BMI divided into three groups according to the WHO-classification, underweight/normal weight (<25), pre-obesity (25–29.9), and obesity (> 30) [20]. The underweight group was merged with the normal weight group as the number of underweight women were too few.

### Statistical analyses

We cross-linked data from the two registers using a unique serial number for each woman, guaranteeing anonymity. Patient characteristics are presented as means, or number and per cent, and differences were tested with Chi 2 test. Comparisons between caesarean section and vaginal delivery were made using univariate and multivariable regression analyses for unadjusted and adjusted data respectively. Odds ratio for risk factors was calculated using a multivariable regression model adjusted for all possible risk factors from Table 1.

**Table 1:** Maternal characteristics*.

	Ceasarean n (%)	Vaginal n (%)	*p*-value**
**Total (481 368)**	79 052 (16.4)	402 316 (83.6)	N/A
**Maternal age at delivery**			
Mean -years	30.72	28.03	< 0.001
< 30	38 964 (49.3)	280 242 (69.7)	< 0.001
30–35	24 444 (30.9)	92 941 (23.1)	< 0.001
>35	15 644 (19.8)	29 131 (7.2)	< 0.001
Missing	0	2 (0.0)	
**Mean infant birthweight (g)**	3373	3444	< 0.001
Missing	173 (0.2)	513 (0.1)	
**Smoking (during pregnancy)**	4707 (6.3)	24230 (6.3)	0.851
Missing	4 240 (5.4)	18 322 (4.6)	
**Nr deliveries during study period**	1.44	1.67	< 0.001
**Maternal BMI**			
Mean (kg)	25.30	23.97	< 0.001
< 25	41 603 (57.5)	258 191 (69.4)	< 0.001
25–30	19 363 (26.8)	80 432 (21.6)	< 0.001
>30	11 371 (15.7)	33 494 (9.0)	< 0.001
Missing	6715 (8.5)	30 199 (7.5)	
**Pre-eclampsia/Eclampsia**	8 116 (10.3)	12 450 (3.1)	< 0.001
**Emergency Section**	21290 (26.9)	N/A	N/A

* All data from first delivery, except nr deliveries during study period.

** Chi^2^ -test.

All statistical analyses were performed using the software SPSS Statistics 27 and the SAS software, version 9.4.

## Results

**Fig 1:**
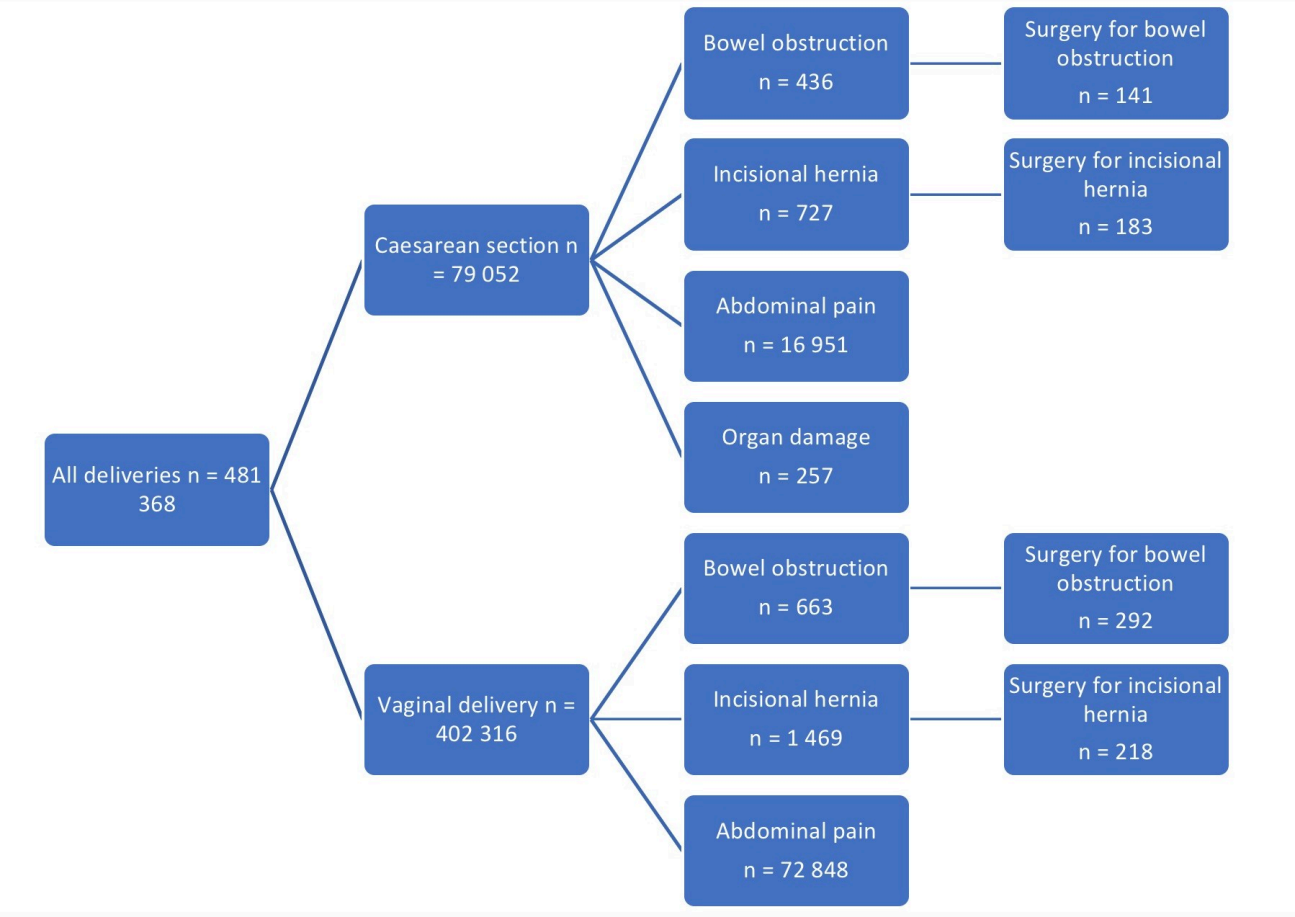
Flow chart of patients included and surgical complications after caesarean section and vaginal delivery controls. Primiparas.

Altogether 79 052 primiparas delivering with caesarean section were included in the study group and 402 316 primiparas delivering vaginally as the control group (Fig 1).

Women in the caesarean group were older, heavier, delivered smaller babies, and had significantly more frequent pre-eclampsia/eclampsia episodes than women in the vaginal delivery group (Table 1).

In the caesarean section group, the risks for all surgical complications were significantly greater than for the controls. For bowel obstruction the OR was 2.92 (CI 2.55–3.34) and for incisional hernia 2.71 (CI 2.46–3.00). Similarly, the risks for surgery due to bowel obstruction and incisional hernia were increased compared to controls; OR 2.12; CI 1.70–2.65 and OR 3.35; CI 2.68–4.18 respectively (see Table 2A).

**Table 2:** a. Odds ratio for surgical complications after delivery. Uni- and multivariable logistic regression analysis. b. Odds ratio for uterine rupture and placenta praevia during pregnancy or delivery. Uni- and multivariable logistic regression analysis.

		No (%)	Unadjusted OR (CI 95%)	*p*-value	Adjusted OR (CI 95%)*†	*p*-value
**Bowel obstruction**	VD	663 (0.2)	1			
	CD	436 (0.6)	3.36 (2.98–3.79)	< 0.001	2.92 (2.55–3.34)	< 0.001
**Surgery for bowel obstruction**	VD	292 (0.1)	1			
	CD	141 (0.2)	2.46 (2.01–3.01)	< 0.001	2.12 (1.70–2.65)	< 0.001
**Incisional hernia**	VD	1469 (0.4)	1			
	CD	727 (1.0)	2.53 (2.32–2.77)	< 0.001	2.71 (2.46–3.00)	<0.001
**Surgery for Incisional hernia**	VD	218 (0.1)	1			
	CD	183 (0.2)	4.28 (3.52–5.21)	< 0.001	3.35 (2.68–4.18)	< 0.001
**Abdominal pain**	VD	72 848 (18.1)	1			
	CD	16 951 (21.4)	1.24 (1.21–1.26)	< 0.001	1.41 (1.38–1.44)	<0.001
**All**	VD	73835 (18.4)	1			
	CD	17489 (22.1)	1.26 (1.24–1.29)	< 0.001	1.44 (1.41–1.47)	< 0.001
**All except abdominal pain**	VD	2088 (0.5)	1		1	
	CD	1135 (1.4)	4.04 (3.79–4.31)	< 0.001	2.81 (2.59–3.05)	< 0.001
**Uterine rupture**	VD	75 (0.00)	1			
	CD	566 (0.70)	38.68 (30.40–49.22)	< 0.001	55.10 (42.44–71.54)	<0.001
**Placenta praevia**	VD	98 (0.02)	1		1	
	CD	1353 (0.28)	68.15 (55.78–83.28)	<0.001	67.72 (54.68–83.89)	<0.001

* Adjusted for birthweigh (median total group = 3450), smoking, nr deliveries, BMI, maternal age and pre-eclampsia/eclampsia.

† Missing cases in the adjusted group = 41355 (8.6%) due to missing data for one or more of the variables adjusted for.

When analysing surgical complications occurring within 42 days after caesarean section, figures were low, with bleeding (n = 203, 0.17%), organ damage (n = 257, 0.22%), and wound dehiscence (n = 272, 0.23%) occurring in less than 0.5%. Infection was the most common complication; 813 of 118 057 (0, 69%) (Table 3).

**Table 3:** Complications within 42 days after caesarean section.

	Single birth	Multiple birth	Tot 118 057
**Bleeding**	203 (0.17)	10 (0.01)	213 (0.18)
**Infection**	785 (0.66)	28 (0.02)	813 (0.69)
**Organ damage**	252 (0.21)	5 (0.00)	257 (0.22)
**Wound dehiscence**	259 (0.22)	13 (0.01)	272 (0.23)
**Bowel obstruction**	100 (0.08)	9 (0.01)	109 (0.09)
**Other**	63 (0.05)	1 (0.00)	64 (0.05)

For abdominal pain, which was the most common complication, there was a significant difference in risk, with 21.4% (OR 1.24; CI 1.21–1.26) receiving a diagnosis for abdominal pain after caesarean section and 18.1% after vaginal delivery.

Separate risk factor analyses for abdominal pain and separate age groups showed that the youngest group (18–25 years) were most frequently diagnosed with abdominal pain.

Smoking, obesity, and one or more previous sections significantly increased the risk for almost all surgical complications. Emergency section was associated with increased risk for surgery due to bowel obstruction or hernia, but not for other complications (Table 4).

**Table 4:** Multivariable logistic regression analysis: Odds ratio for surgical complication after caesarean section*.

Complication	Parameter	OR (95%CI)	*p*-value
**Bowel obstruction**	Emergency section	1.18 (0.94–1.49)	0.163
	Birthweight > Median	0.67 (0.54–0.84)	< 0.001
	Smoking	1.91 (1.37–2.65)	< 0.001
	>1 Delivery	1.42 (1.15–1.76)	0.001
	BMI 25.0–29.9	0.90 (0.68–1.19)	0.897
	BMI ≥ 30	2.37 (1.85–3.05)	< 0.001
	Maternal age 30–34	1.03 (0.81–1.32)	0.811
	Maternal age ≥ 35	0.98 (0.74–1.29)	0.882
	Pre-eclampsia/Eclampsia	0.86 (0.60–1.21)	0.378
**Incisional hernia**	Emergency section	1.18 (0.94–1.49)	0.163
	Birthweight > Median	0.67 (0.54–0.83)	< 0.001
	Smoking	1.91 (1.37–2.65)	0.001
	>1 Delivery	1.42 (1.15–1.76)	0.001
	BMI 25–30	0.90 (0.68–1.19)	0.449
	BMI > 30	2.37 (1.85–3.05)	< 0.001
	Maternal age 30–35	1.03 (0.81–1.32)	0.811
	Maternal age > 35	0.98 (0.74–1.629)	0.882
	Pre-eclampsia/Eclampsia	0.86 (0.61–1.21)	0.278
**Surgery for bowel obstruction or hernia**	Emergency section	1.41 (1.08–1.84)	< 0.011
	Birthweight > Median	0.73 (0.57–0.94)	0.016
	Smoking	2.16 (1.49–3.13)	< 0.001
	>1 Delivery	2.31 (1.80–2.97)	< 0.001
	BMI 25–30	1.20 (0.86–1.69)	0.286
	BMI > 30	4.32 (3.25–5.74)	< 0.001
	Maternal age 30–35	1.22 (0.92–1.62)	0.178
	Maternal age > 35	1.15 (0.83–1.58)	0.414
	Pre-eclampsia/Eclampsia	1.01 (0.70–1.47)	0.951
**Abdominal pain**	Emergency section	1.01 (0.97–1.05)	0.705
	Birthweight > Median	0.93 (0.89–0.97)	< 0.001
	Smoking	1.64 (1.53–1.76)	< 0.001
	>1 Delivery	1.54 (1.48–1.60)	< 0.001
	BMI 25–30	1.10 (1.06–1.15)	< 0.001
	BMI > 30	1.39 (1.32–1.46)	< 0.001
	Maternal age 30–35	0.67 (0.65–0.70)	< 0.001
	Maternal age > 35	0.64 (0.61–0.67)	< 0.001
	Pre-eclampsia/Eclampsia	0.95 (0.89–1.01)	0.101

*11939 observations (15.1%) were not included in the multiple logistic regressions due to missing data for any of the risk factors included.

†Median birthweight caesarean = 3440.

The median time from delivery to complication diagnosis after a caesarean section was 3.2 years for bowel obstruction, 4.1 years for incisional hernia, and 2.9 years for abdominal pain.

**Fig 2:**
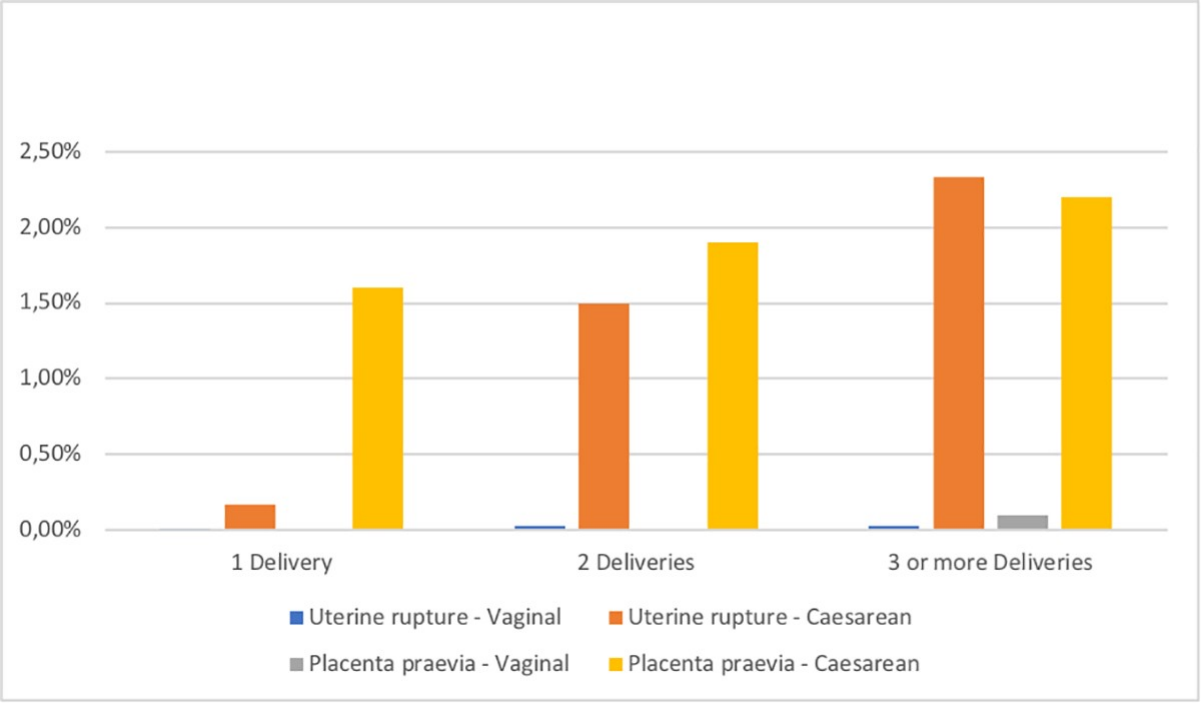
Risk in per cent for uterine rupture and placenta praevia after 1, 2 or 3 or more deliveries.

The risk for placenta praevia and uterine rupture increased after caesarean section (Table 2B) and the risk increased with each caesarean section (Fig 2).

## Discussion

The main findings in this population-based study are that caesarean section is associated with a significantly higher risk for all surgical complications investigated compared to a control group of vaginal deliveries only. For bowel obstruction and incisional hernia, the risk was increased threefold in the caesarean section group, with many patients needing surgery. Obesity, smoking, and one or more previous sections are significant risk factors for these complications. Compared to elective caesarean section, emergency section increases the risk for bowel obstruction and incisional hernia.

When choosing method of delivery, it is important to take into consideration that caesarean section is an abdominal procedure with all the accompanying risks for complications such as bowel obstruction, incisional hernia, organ damage, and abdominal pain.

The risk for incisional hernia is present after all forms of abdominal surgery. A newly published systematic review reports the rate of incisional hernia to be 0.0–5.6% after caesarean section. A possible reason for the wide range is the large number of midline incisions in some developing countries at the time of the report [11]. Two larger studies in the review were conducted in high-income countries where transverse incision was recommended to limit the occurrence of incisional hernia [21]. These showed incisional hernia repair rates of 0.16% [22] and 0.5 [23] %. The rate of 1.0% in the present study is comparable, though difference in study design makes it difficult to compare results.

The bowel obstruction rate after caesarean section in this study was higher than that reported by Andhoff *et al* [10] (0.6% vs 0.2%). One possible explanation may be that women with comorbidity and previous abdominal surgery were not excluded from the present study.

The actual number of complications is relatively low, which is to be expected as the cohort consists of young, mostly healthy women with low risk for complications after surgery [24]. There may be some under-reporting, which is always a problem regarding complications, but there is no reason to believe that this should differ between the caesarean section and control groups. However, even a low complication rate must be taken into consideration in view of the great number of caesarean sections performed each year throughout the world. It is also obvious that with multiple cesarean sections, the risk of complications increases even more.

Obesity and smoking were significant risk factors for almost all the surgical complications investigated. Smoking has been shown to increase the risk for complications after many surgical procedures but previously not after caesarean sections [25,26]. Although maybe not surprising, this is alarming, since both obesity and smoking are growing problems among women in many parts of the world [27,28].

Previous studies have shown damage to the bladder in 0.03–1% and damage to the ureters in 0.02–0.05% in connection with caesarian section [7,29]. Bowel damage is reported in less than 0.1%. Although most studies are small and/or performed in settings with varying standards of obstetric care, they are in line with the 0.22% organ damage in the caesarean section group in the present study.

The incidence of wound dehiscence has hardly been studied. Otkjaer *et al* showed an incidence of 0.19–0.25% in a Danish register-based cohort study [29]. The present study shows a similar figure of 272 of 118 057 (0.23%).

Abdominal pain was the most common complication after both caesarean section and vaginal delivery. Liu *et al* showed that 7.8% had persistent abdominal pain 2 months after caesarean delivery which had fallen to 1.1% after one year [30]. The numbers in this study were much higher, 21.4% after caesarean section and 18.1% after vaginal delivery, with a median time to diagnosis of 2.9 years after caesarean section. Abdominal pain, however, is multifactorial and associated with many confounders and conditions. Contrary to all other complications it is more frequent among younger women.

Placenta praevia and uterine rupture were incidental findings in our material. Though barely classified as surgical complications, they often need surgery when diagnosed [31,32]. Comparison between caesarean section and vaginal delivery regarding these diagnoses is not fair since these complications arise from a previous delivery and often ends with caesarean section with or without hysterectomy. With our study design, causality for these complications could be reversed. There might also be some placenta praevias or uterine ruptures that could have occurred during a vaginal delivery and required caesarean section, but such cases were excluded from this study. What is interesting, however, is the increase in risk for placenta praevia and uterine rupture after each new caesarean section as shown in Fig 2.

The main strength of this study is that it is based on nationwide registers with almost 100% coverage, providing a large unselected population that enabled us to study rare surgical events as well as allowing generalisation of results and minimal risk for selection bias. Another strength is the large control group of vaginal deliveries. Complications can occur regardless of the method of delivery and the cause of complication in each case is impossible to establish in materials of this size. A control group not including caesarean sections is fundamental to evaluate the relative risk of this abdominal procedure. Another strength is that as we can follow individuals over time, we see that the complications do not occur directly after delivery but after several years.

There are also obvious limitations with this study design. The group of women who delivered by caesarean section for medical reasons are possibly at higher risk for complications from the beginning (and were therefore planned for section or needed an emergency section). Although we adjusted for several possible confounders, there could still be residual confounders not accounted for in present study such as maternal comorbidity. The observational design of this study could result in reversed causality in some cases. Women with previous surgery were not excluded but the same was the case for the control group of vaginal deliveries. Furthermore, the surgical complications investigated in the study may be the result of previous abdominal surgery, but compared to the control group, the increased complication rates in the caesarean section group cannot be denied.

The years leading up to 2005 were not included as the amount of missing data in the registers at that time was too high. Since complications can develop many years after abdominal surgery, it is reasonable to assume that the number of complications should increase if the follow-up time is longer. Furthermore, Swedish primary care does not report to the National Patient Register and thus women seeking primary care are not included. Minor complications in hospital and outpatient care, are known to be under-reported in the registers, whereas more severe complications, such as bowel obstruction and surgery, require at least specialist outpatient care, and these numbers are more reliable [16].

Almost half of the world’s population gives birth at least once. It is therefore most important to be aware of the advantages and disadvantages of each delivery method. Present study reports on surgical complications after cesarean section while a large number of previous studies have reported long-term consequences of vaginal delivery such as urinary and faecal incontinence [33,34], pelvic organ prolapse and pain conditions [35,36].

## Conclusion

The number of women who choose to give birth by caesarean section without medical indication is steadily increasing. This study shows a substantially increased risk for surgical complications after caesarean section compared to vaginal delivery, and several of these complications may continue to affect women later in life. More than one section, smoking and obesity are significant risk factors. Risks and risk factors must be taken into consideration when planning mode of delivery, and this is essential in promoting the health of women worldwide.

## Supporting information

S1 Table Diagnosis codes used in the study. (DOCX)

S2 Table Diagnosis codes used in the study. Short-term complications.
(DOCX)

S1 File (RTF)
