# Supplementary material for: Surgical complications after caesarean section: A population-based cohort study
Source: PLoS One. 2021 Oct 5;16(10):e0258222. doi: 10.1371/journal.pone.0258222 (PMC8491947; doi:10.1371/journal.pone.0258222)
Supplement: S1 Table — (DOCX) [file pone.0258222.s001.docx]

| **S 1. Table** Diagnosis codes used in the study. | |
| --- | --- |
|  |  |
| Bowel obstruction | K56 ( not K56.2-3) |
|  |  |
| Surgery for bowel obstruction | JAH00-01, JFA, JFB, JFK, JFL* |
|  |  |
| Incisional hernia | K43 |
|  |  |
| Surgery for incisional hernia | JAD**†** |
|  |  |
| Abdominal pain | R10 |
|  |  |
| Uterine rupture | O71.0-1 |
|  |  |
| *in combination with K56  **†** in combination with K43 | |
