# Supplementary material for: Surgical complications after caesarean section: A population-based cohort study
Source: PLoS One. 2021 Oct 5;16(10):e0258222. doi: 10.1371/journal.pone.0258222 (PMC8491947; doi:10.1371/journal.pone.0258222)
Supplement: S2 Table — Short-term complications. (DOCX) [file pone.0258222.s002.docx]

| **S 2.** **Table** Diagnosis codes used in the study. Short-term complications. | | |
| --- | --- | --- |
|  |  |  |
| Bleeding | T81.0 |  |
|  | T81.7 |  |
|  | S35 |  |
|  |  |  |
| Infection | T81.4 |  |
|  |  |  |
| Organ damage | T81.2 |  |
|  | T81.8A |  |
|  | S36 |  |
|  | S37.0-7 |  |
|  |  |  |
| Wound rupture | T81.3 |  |
|  | O90.0 |  |
|  |  |  |
| Other | T81.1 | Postprocedural shock |
|  | T81.5 | Complications of foreign body accidentally left in abdomen during procedure |
|  | T81.6 | Acute reaction to foreign substance accidentally left during procedure |
|  | T81.8 | Other complication of procedure |
|  | T81.9 | Unspecified complication of procedure |
|  |  |  |
