# Supplementary material for: Surgical complications after caesarean section: A population-based cohort study
Source: PLoS One. 2021 Oct 5;16(10):e0258222. doi: 10.1371/journal.pone.0258222 (PMC8491947; doi:10.1371/journal.pone.0258222)
Supplement: S1 File — (RTF) [file pone.0258222.s003.rtf]

Data from SPSS.

Variables used in the logistic regression models:
Ileus – Bowel obstruction
Hernia – Incisional hernia
Op_ileus – Surgery for bowel obstruction
Op_hernia – Surgery for incisional hernia
Any_op – Surgery for bowel obstruction or incisional hernia
Ab_pain – Abdominal pain
Any_diagnosis – All (bowel obstruction, incisional hernia or abdominal pain)
All_but_ab – All diagnosis except abdominal pain (bowel obstruction or incisional hernia)
Ut_rupture_tot – Uterine rupture
Pl_previa_tot – Placenta previa
Sectio – Caesarean section
ELEK/AKUT – Emergency section
BVIKT_median_alla – Above median birthweight total (3450g)
BVIKT_med_CS – Above median birthweight caesarean (3440g)
Roker – Smoking
Morethan1_del – More than 1 delivery
BMI_3_gr - BMI divided in 3 cathegories
Ålder_3_gr – Age in 3 cathegories
Pre_ecl_cat – Preeclampsia/Eclampsia

TABLE 4. Logistic regression

Bowel obstruction

Logistic Regression

Case Processing Summary	
Unweighted Casesa	N	Percent	
Selected Cases	Included in Analysis	67113	84.9	
	Missing Cases	11939	15.1	
	Total	79052	100.0	
Unselected Cases	0	.0	
Total	79052	100.0	

a. If weight is in effect, see classification table for the total number of cases.	


Dependent Variable Encoding	
Original Value	Internal Value	
0	0	
1	1	


Categorical Variables Codings	
	Frequency	Parameter coding	
		(1)	(2)	
Ålder_3_gr	1.00	27984	.000	.000	
	2.00	22338	1.000	.000	
	3.00	16791	.000	1.000	
BMI_3_gr	1.00	38848	.000	.000	
	2.00	17879	1.000	.000	
	3.00	10386	.000	1.000	


Block 0: Beginning Block


Classification Tablea,b	
	Observed	Predicted	
		ileus	Percentage Correct	
		0	1		
Step 0	ileus	0	66766	0	100.0	
		1	347	0	.0	
	Overall Percentage			99.5	

a. Constant is included in the model.	
b. The cut value is .500	


Variables in the Equation	
	B	S.E.	Wald	df	Sig.	Exp(B)	
Step 0	Constant	-5.260	.054	9549.654	1	.000	.005	


Block 1: Method = Enter


Omnibus Tests of Model Coefficients	
	Chi-square	df	Sig.	
Step 1	Step	87.011	9	.000	
	Block	87.011	9	.000	
	Model	87.011	9	.000	


Model Summary	
Step	-2 Log likelihood	Cox & Snell R Square	Nagelkerke R Square	
1	4258.969a	.001	.021	

a. Estimation terminated at iteration number 8 because parameter estimates changed by less than .001.	


Classification Tablea	
	Observed	Predicted	
		ileus	Percentage Correct	
		0	1		
Step 1	ileus	0	66766	0	100.0	
		1	347	0	.0	
	Overall Percentage			99.5	

a. The cut value is .500	


Variables in the Equation	
	B	S.E.	Wald	df	Sig.	Exp(B)	95% C.I.for EXP(B)	
							Lower	Upper	
Step 1a	ELEKAKUT	.165	.118	1.950	1	.163	1.179	.936	1.487	
	B_vikt_med_CS	-.404	.113	12.843	1	.000	.668	.535	.833	
	roker	.645	.169	14.621	1	.000	1.906	1.369	2.652	
	Morethan1_del	.352	.109	10.325	1	.001	1.422	1.147	1.762	
	BMI_3_gr			55.930	2	.000				
	BMI_3_gr(1)	-.108	.143	.573	1	.449	.897	.677	1.188	
	BMI_3_gr(2)	.864	.128	45.612	1	.000	2.372	1.846	3.048	
	Ålder_3_gr			.127	2	.938				
	Ålder_3_gr(1)	.030	.125	.057	1	.811	1.030	.806	1.318	
	Ålder_3_gr(2)	-.021	.141	.022	1	.882	.979	.744	1.290	
	pre_ecl_cat	-.156	.177	.777	1	.378	.855	.604	1.211	
	Constant	-5.481	.134	1668.768	1	.000	.004			

a. Variable(s) entered on step 1: ELEKAKUT, B_vikt_med_CS, roker, Morethan1_del, BMI_3_gr, Ålder_3_gr, pre_ecl_cat.	

Hernia


Logistic Regression


Case Processing Summary	
Unweighted Casesa	N	Percent	
Selected Cases	Included in Analysis	67113	84.9	
	Missing Cases	11939	15.1	
	Total	79052	100.0	
Unselected Cases	0	.0	
Total	79052	100.0	

a. If weight is in effect, see classification table for the total number of cases.	


Dependent Variable Encoding	
Original Value	Internal Value	
0	0	
1	1	


Categorical Variables Codings	
	Frequency	Parameter coding	
		(1)	(2)	
Ålder_3_gr	1.00	27984	.000	.000	
	2.00	22338	1.000	.000	
	3.00	16791	.000	1.000	
BMI_3_gr	1.00	38848	.000	.000	
	2.00	17879	1.000	.000	
	3.00	10386	.000	1.000	


Block 0: Beginning Block


Classification Tablea,b	
	Observed	Predicted	
		hernia	Percentage Correct	
		0	1		
Step 0	hernia	0	66526	0	100.0	
		1	587	0	.0	
	Overall Percentage			99.1	

a. Constant is included in the model.	
b. The cut value is .500	


Variables in the Equation	
	B	S.E.	Wald	df	Sig.	Exp(B)	
Step 0	Constant	-4.730	.041	13019.807	1	.000	.009	


Block 1: Method = Enter


Omnibus Tests of Model Coefficients	
	Chi-square	df	Sig.	
Step 1	Step	222.267	9	.000	
	Block	222.267	9	.000	
	Model	222.267	9	.000	


Model Summary	
Step	-2 Log likelihood	Cox & Snell R Square	Nagelkerke R Square	
1	6510.297a	.003	.035	

a. Estimation terminated at iteration number 8 because parameter estimates changed by less than .001.	


Classification Tablea	
	Observed	Predicted	
		hernia	Percentage Correct	
		0	1		
Step 1	hernia	0	66526	0	100.0	
		1	587	0	.0	
	Overall Percentage			99.1	

a. The cut value is .500	


Variables in the Equation	
	B	S.E.	Wald	df	Sig.	Exp(B)	95% C.I.for EXP(B)	
							Lower	Upper	
Step 1a	ELEKAKUT	.125	.093	1.830	1	.176	1.134	.945	1.360	
	B_vikt_med_CS	.348	.087	15.987	1	.000	1.416	1.194	1.678	
	roker	.435	.151	8.241	1	.004	1.544	1.148	2.078	
	Morethan1_del	1.109	.087	162.120	1	.000	3.030	2.555	3.594	
	BMI_3_gr			34.056	2	.000				
	BMI_3_gr(1)	-.044	.104	.181	1	.671	.957	.781	1.173	
	BMI_3_gr(2)	.560	.104	28.797	1	.000	1.750	1.427	2.147	
	Ålder_3_gr			7.942	2	.019				
	Ålder_3_gr(1)	.025	.098	.063	1	.801	1.025	.845	1.243	
	Ålder_3_gr(2)	.281	.106	7.059	1	.008	1.324	1.076	1.629	
	pre_ecl_cat	-.155	.145	1.149	1	.284	.856	.645	1.137	
	Constant	-5.706	.117	2366.945	1	.000	.003			

a. Variable(s) entered on step 1: ELEKAKUT, B_vikt_med_CS, roker, Morethan1_del, BMI_3_gr, Ålder_3_gr, pre_ecl_cat.	


Surgery for bowel obstruction or hernia


Logistic Regression


Case Processing Summary	
Unweighted Casesa	N	Percent	
Selected Cases	Included in Analysis	67113	84.9	
	Missing Cases	11939	15.1	
	Total	79052	100.0	
Unselected Cases	0	.0	
Total	79052	100.0	

a. If weight is in effect, see classification table for the total number of cases.	


Dependent Variable Encoding	
Original Value	Internal Value	
0	0	
1	1	


Categorical Variables Codings	
	Frequency	Parameter coding	
		(1)	(2)	
Ålder_3_gr	1.00	27984	.000	.000	
	2.00	22338	1.000	.000	
	3.00	16791	.000	1.000	
BMI_3_gr	1.00	38848	.000	.000	
	2.00	17879	1.000	.000	
	3.00	10386	.000	1.000	


Block 0: Beginning Block


Classification Tablea,b	
	Observed	Predicted	
		any_op	Percentage Correct	
		0	1		
Step 0	any_op	0	66858	0	100.0	
		1	255	0	.0	
	Overall Percentage			99.6	

a. Constant is included in the model.	
b. The cut value is .500	


Variables in the Equation	
	B	S.E.	Wald	df	Sig.	Exp(B)	
Step 0	Constant	-5.569	.063	7878.638	1	.000	.004	


Block 1: Method = Enter


Omnibus Tests of Model Coefficients	
	Chi-square	df	Sig.	
Step 1	Step	166.751	9	.000	
	Block	166.751	9	.000	
	Model	166.751	9	.000	


Model Summary	
Step	-2 Log likelihood	Cox & Snell R Square	Nagelkerke R Square	
1	3184.442a	.002	.051	

a. Estimation terminated at iteration number 9 because parameter estimates changed by less than .001.	


Classification Tablea	
	Observed	Predicted	
		any_op	Percentage Correct	
		0	1		
Step 1	any_op	0	66858	0	100.0	
		1	255	0	.0	
	Overall Percentage			99.6	

a. The cut value is .500	


Variables in the Equation	
	B	S.E.	Wald	df	Sig.	Exp(B)	95% C.I.for EXP(B)	
							Lower	Upper	
Step 1a	ELEKAKUT	.344	.136	6.416	1	.011	1.411	1.081	1.841	
	B_vikt_med_CS	-.314	.131	5.764	1	.016	.730	.565	.944	
	roker	.769	.189	16.492	1	.000	2.158	1.489	3.129	
	Morethan1_del	.837	.128	42.541	1	.000	2.310	1.796	2.972	
	BMI_3_gr			115.119	2	.000				
	BMI_3_gr(1)	.184	.173	1.140	1	.286	1.202	.857	1.686	
	BMI_3_gr(2)	1.464	.145	102.059	1	.000	4.322	3.254	5.741	
	Ålder_3_gr			1.897	2	.387				
	Ålder_3_gr(1)	.197	.146	1.818	1	.178	1.217	.915	1.621	
	Ålder_3_gr(2)	.135	.166	.667	1	.414	1.145	.828	1.584	
	pre_ecl_cat	.012	.191	.004	1	.951	1.012	.696	1.471	
	Constant	-6.534	.175	1391.528	1	.000	.001			

a. Variable(s) entered on step 1: ELEKAKUT, B_vikt_med_CS, roker, Morethan1_del, BMI_3_gr, Ålder_3_gr, pre_ecl_cat.	


Abdominal Pain


Logistic Regression

Case Processing Summary	
Unweighted Casesa	N	Percent	
Selected Cases	Included in Analysis	67113	84.9	
	Missing Cases	11939	15.1	
	Total	79052	100.0	
Unselected Cases	0	.0	
Total	79052	100.0	

a. If weight is in effect, see classification table for the total number of cases.	


Dependent Variable Encoding	
Original Value	Internal Value	
0	0	
1	1	


Categorical Variables Codings	
	Frequency	Parameter coding	
		(1)	(2)	
Ålder_3_gr	1.00	27984	.000	.000	
	2.00	22338	1.000	.000	
	3.00	16791	.000	1.000	
BMI_3_gr	1.00	38848	.000	.000	
	2.00	17879	1.000	.000	
	3.00	10386	.000	1.000	


Block 0: Beginning Block


Classification Tablea,b	
	Observed	Predicted	
		ab_pain	Percentage Correct	
		0	1		
Step 0	ab_pain	0	52968	0	100.0	
		1	14145	0	.0	
	Overall Percentage			78.9	

a. Constant is included in the model.	
b. The cut value is .500	


Variables in the Equation	
	B	S.E.	Wald	df	Sig.	Exp(B)	
Step 0	Constant	-1.320	.009	19461.339	1	.000	.267	


Block 1: Method = Enter


Omnibus Tests of Model Coefficients	
	Chi-square	df	Sig.	
Step 1	Step	1455.271	9	.000	
	Block	1455.271	9	.000	
	Model	1455.271	9	.000	


Model Summary	
Step	-2 Log likelihood	Cox & Snell R Square	Nagelkerke R Square	
1	67666.699a	.021	.033	

a. Estimation terminated at iteration number 4 because parameter estimates changed by less than .001.	


Classification Tablea	
	Observed	Predicted	
		ab_pain	Percentage Correct	
		0	1		
Step 1	ab_pain	0	52968	0	100.0	
		1	14145	0	.0	
	Overall Percentage			78.9	

a. The cut value is .500	


Variables in the Equation	
	B	S.E.	Wald	df	Sig.	Exp(B)	95% C.I.for EXP(B)	
							Lower	Upper	
Step 1a	ELEKAKUT	.008	.022	.143	1	.705	1.008	.966	1.052	
	B_vikt_med_CS	-.074	.020	14.100	1	.000	.929	.893	.965	
	roker	.496	.036	193.432	1	.000	1.643	1.532	1.762	
	Morethan1_del	.432	.020	491.314	1	.000	1.541	1.483	1.601	
	BMI_3_gr			152.124	2	.000				
	BMI_3_gr(1)	.098	.023	18.669	1	.000	1.103	1.055	1.153	
	BMI_3_gr(2)	.327	.027	151.601	1	.000	1.387	1.317	1.462	
	Ålder_3_gr			474.261	2	.000				
	Ålder_3_gr(1)	-.395	.022	314.027	1	.000	.674	.645	.704	
	Ålder_3_gr(2)	-.454	.025	324.180	1	.000	.635	.605	.667	
	pre_ecl_cat	-.052	.032	2.696	1	.101	.949	.892	1.010	
	Constant	-1.341	.023	3406.119	1	.000	.262			

a. Variable(s) entered on step 1: ELEKAKUT, B_vikt_med_CS, roker, Morethan1_del, BMI_3_gr, Ålder_3_gr, pre_ecl_cat.	


TABLE 2a-b, adjusted odds ratio

Bowel obstruction


Logistic Regression


Case Processing Summary	
Unweighted Casesa	N	Percent	
Selected Cases	Included in Analysis	440013	91.4	
	Missing Cases	41355	8.6	
	Total	481368	100.0	
Unselected Cases	0	.0	
Total	481368	100.0	

a. If weight is in effect, see classification table for the total number of cases.	


Dependent Variable Encoding	
Original Value	Internal Value	
0	0	
1	1	


Categorical Variables Codings	
	Frequency	Parameter coding	
		(1)	(2)	
Ålder_3_gr	1.00	259634	.000	.000	
	2.00	126164	1.000	.000	
	3.00	54215	.000	1.000	
BMI_3_gr	1.00	296686	.000	.000	
	2.00	98910	1.000	.000	
	3.00	44417	.000	1.000	


Block 0: Beginning Block


Classification Tablea,b	
	Observed	Predicted	
		ileus	Percentage Correct	
		0	1		
Step 0	ileus	0	439022	0	100.0	
		1	991	0	.0	
	Overall Percentage			99.8	

a. Constant is included in the model.	
b. The cut value is .500	


Variables in the Equation	
	B	S.E.	Wald	df	Sig.	Exp(B)	
Step 0	Constant	-6.094	.032	36714.780	1	.000	.002	


Block 1: Method = Enter


Omnibus Tests of Model Coefficients	
	Chi-square	df	Sig.	
Step 1	Step	688.660	9	.000	
	Block	688.660	9	.000	
	Model	688.660	9	.000	


Model Summary	
Step	-2 Log likelihood	Cox & Snell R Square	Nagelkerke R Square	
1	13373.072a	.002	.050	

a. Estimation terminated at iteration number 9 because parameter estimates changed by less than .001.	


Classification Tablea	
	Observed	Predicted	
		ileus	Percentage Correct	
		0	1		
Step 1	ileus	0	439022	0	100.0	
		1	991	0	.0	
	Overall Percentage			99.8	

a. The cut value is .500	


Variables in the Equation	
	B	S.E.	Wald	df	Sig.	Exp(B)	95% C.I.for EXP(B)	
							Lower	Upper	
Step 1a	Sectio	1.071	.069	237.992	1	.000	2.918	2.546	3.343	
	BVIKT_median_alla	-.278	.065	18.362	1	.000	.757	.667	.860	
	roker	.580	.100	33.899	1	.000	1.786	1.469	2.170	
	Morethan1_del	.392	.066	35.138	1	.000	1.480	1.300	1.684	
	BMI_3_gr			387.533	2	.000				
	BMI_3_gr(1)	.204	.085	5.770	1	.016	1.227	1.038	1.449	
	BMI_3_gr(2)	1.427	.075	363.466	1	.000	4.164	3.596	4.822	
	Ålder_3_gr			3.493	2	.174				
	Ålder_3_gr(1)	-.039	.076	.269	1	.604	.961	.829	1.115	
	Ålder_3_gr(2)	.145	.093	2.443	1	.118	1.156	.964	1.386	
	pre_ecl_cat	-.095	.124	.580	1	.446	.910	.713	1.160	
	Constant	-6.839	.078	7593.268	1	.000	.001			

a. Variable(s) entered on step 1: Sectio, BVIKT_median_alla, roker, Morethan1_del, BMI_3_gr, Ålder_3_gr, pre_ecl_cat.	

Surgery for bowel obstruction


Logistic Regression


Case Processing Summary	
Unweighted Casesa	N	Percent	
Selected Cases	Included in Analysis	440013	91.4	
	Missing Cases	41355	8.6	
	Total	481368	100.0	
Unselected Cases	0	.0	
Total	481368	100.0	

a. If weight is in effect, see classification table for the total number of cases.	


Dependent Variable Encoding	
Original Value	Internal Value	
0	0	
1	1	


Categorical Variables Codings	
	Frequency	Parameter coding	
		(1)	(2)	
Ålder_3_gr	1.00	259634	.000	.000	
	2.00	126164	1.000	.000	
	3.00	54215	.000	1.000	
BMI_3_gr	1.00	296686	.000	.000	
	2.00	98910	1.000	.000	
	3.00	44417	.000	1.000	


Block 0: Beginning Block


Classification Tablea,b	
	Observed	Predicted	
		op_ileus	Percentage Correct	
		0	1		
Step 0	op_ileus	0	439617	0	100.0	
		1	396	0	.0	
	Overall Percentage			99.9	

a. Constant is included in the model.	
b. The cut value is .500	


Variables in the Equation	
	B	S.E.	Wald	df	Sig.	Exp(B)	
Step 0	Constant	-7.012	.050	19454.421	1	.000	.001	


Block 1: Method = Enter


Omnibus Tests of Model Coefficients	
	Chi-square	df	Sig.	
Step 1	Step	396.306	9	.000	
	Block	396.306	9	.000	
	Model	396.306	9	.000	


Model Summary	
Step	-2 Log likelihood	Cox & Snell R Square	Nagelkerke R Square	
1	5949.749a	.001	.063	

a. Estimation terminated at iteration number 10 because parameter estimates changed by less than .001.	


Classification Tablea	
	Observed	Predicted	
		op_ileus	Percentage Correct	
		0	1		
Step 1	op_ileus	0	439617	0	100.0	
		1	396	0	.0	
	Overall Percentage			99.9	

a. The cut value is .500	


Variables in the Equation	
	B	S.E.	Wald	df	Sig.	Exp(B)	95% C.I.for EXP(B)	
							Lower	Upper	
Step 1a	Sectio	.751	.114	43.462	1	.000	2.119	1.695	2.649	
	BVIKT_median_alla	-.344	.103	11.238	1	.001	.709	.580	.867	
	roker	.790	.141	31.549	1	.000	2.204	1.673	2.904	
	Morethan1_del	.482	.105	21.044	1	.000	1.619	1.318	1.990	
	BMI_3_gr			315.263	2	.000				
	BMI_3_gr(1)	.517	.139	13.799	1	.000	1.677	1.277	2.202	
	BMI_3_gr(2)	2.006	.117	294.923	1	.000	7.434	5.913	9.347	
	Ålder_3_gr			1.155	2	.561				
	Ålder_3_gr(1)	-.128	.121	1.114	1	.291	.880	.693	1.116	
	Ålder_3_gr(2)	-.073	.159	.210	1	.647	.930	.681	1.269	
	pre_ecl_cat	-.255	.207	1.516	1	.218	.775	.516	1.163	
	Constant	-7.917	.129	3765.555	1	.000	.000			

a. Variable(s) entered on step 1: Sectio, BVIKT_median_alla, roker, Morethan1_del, BMI_3_gr, Ålder_3_gr, pre_ecl_cat.	

Hernia


Logistic Regression


Case Processing Summary	
Unweighted Casesa	N	Percent	
Selected Cases	Included in Analysis	440013	91.4	
	Missing Cases	41355	8.6	
	Total	481368	100.0	
Unselected Cases	0	.0	
Total	481368	100.0	

a. If weight is in effect, see classification table for the total number of cases.	


Dependent Variable Encoding	
Original Value	Internal Value	
0	0	
1	1	


Categorical Variables Codings	
	Frequency	Parameter coding	
		(1)	(2)	
Ålder_3_gr	1.00	259634	.000	.000	
	2.00	126164	1.000	.000	
	3.00	54215	.000	1.000	
BMI_3_gr	1.00	296686	.000	.000	
	2.00	98910	1.000	.000	
	3.00	44417	.000	1.000	


Block 0: Beginning Block


Classification Tablea,b	
	Observed	Predicted	
		hernia	Percentage Correct	
		0	1		
Step 0	hernia	0	438044	0	100.0	
		1	1969	0	.0	
	Overall Percentage			99.6	

a. Constant is included in the model.	
b. The cut value is .500	


Variables in the Equation	
	B	S.E.	Wald	df	Sig.	Exp(B)	
Step 0	Constant	-5.405	.023	57260.636	1	.000	.004	


Block 1: Method = Enter


Omnibus Tests of Model Coefficients	
	Chi-square	df	Sig.	
Step 1	Step	828.264	9	.000	
	Block	828.264	9	.000	
	Model	828.264	9	.000	


Model Summary	
Step	-2 Log likelihood	Cox & Snell R Square	Nagelkerke R Square	
1	24402.651a	.002	.034	

a. Estimation terminated at iteration number 9 because parameter estimates changed by less than .001.	


Classification Tablea	
	Observed	Predicted	
		hernia	Percentage Correct	
		0	1		
Step 1	hernia	0	438044	0	100.0	
		1	1969	0	.0	
	Overall Percentage			99.6	

a. The cut value is .500	


Variables in the Equation	
	B	S.E.	Wald	df	Sig.	Exp(B)	95% C.I.for EXP(B)	
							Lower	
Step 1a	Sectio	.998	.051	382.757	1	.000	2.713	2.455	
	BVIKT_median_alla	.333	.046	51.731	1	.000	1.396	1.274	
	roker	.437	.082	28.580	1	.000	1.549	1.319	
	Morethan1_del	.949	.051	347.684	1	.000	2.583	2.338	
	BMI_3_gr			68.853	2	.000			
	BMI_3_gr(1)	-.119	.058	4.190	1	.041	.888	.792	
	BMI_3_gr(2)	.464	.064	53.287	1	.000	1.590	1.404	
	Ålder_3_gr			24.873	2	.000			
	Ålder_3_gr(1)	.040	.053	.587	1	.444	1.041	.939	
	Ålder_3_gr(2)	.331	.067	24.314	1	.000	1.392	1.221	
	pre_ecl_cat	-.057	.098	.333	1	.564	.945	.779	
	Constant	-6.534	.061	11379.553	1	.000	.001		

Variables in the Equation	
	95% C.I.for EXP(B)	
	Upper	
Step 1a	Sectio	2.999	
	BVIKT_median_alla	1.528	
	roker	1.818	
	Morethan1_del	2.854	
	BMI_3_gr		
	BMI_3_gr(1)	.995	
	BMI_3_gr(2)	1.800	
	Ålder_3_gr		
	Ålder_3_gr(1)	1.155	
	Ålder_3_gr(2)	1.588	
	pre_ecl_cat	1.146	
	Constant		

a. Variable(s) entered on step 1: Sectio, BVIKT_median_alla, roker, Morethan1_del, BMI_3_gr, Ålder_3_gr, pre_ecl_cat.	

Surgery for hernia

Logistic Regression


Case Processing Summary	
Unweighted Casesa	N	Percent	
Selected Cases	Included in Analysis	440013	91.4	
	Missing Cases	41355	8.6	
	Total	481368	100.0	
Unselected Cases	0	.0	
Total	481368	100.0	

a. If weight is in effect, see classification table for the total number of cases.	


Dependent Variable Encoding	
Original Value	Internal Value	
0	0	
1	1	


Categorical Variables Codings	
	Frequency	Parameter coding	
		(1)	(2)	
Ålder_3_gr	1.00	259634	.000	.000	
	2.00	126164	1.000	.000	
	3.00	54215	.000	1.000	
BMI_3_gr	1.00	296686	.000	.000	
	2.00	98910	1.000	.000	
	3.00	44417	.000	1.000	


Block 0: Beginning Block


Classification Tablea,b	
	Observed	Predicted	
		op_hernia	Percentage Correct	
		0	1		
Step 0	op_hernia	0	439654	0	100.0	
		1	359	0	.0	
	Overall Percentage			99.9	

a. Constant is included in the model.	
b. The cut value is .500	


Variables in the Equation	
	B	S.E.	Wald	df	Sig.	Exp(B)	
Step 0	Constant	-7.110	.053	18135.544	1	.000	.001	


Block 1: Method = Enter


Omnibus Tests of Model Coefficients	
	Chi-square	df	Sig.	
Step 1	Step	414.705	9	.000	
	Block	414.705	9	.000	
	Model	414.705	9	.000	


Model Summary	
Step	-2 Log likelihood	Cox & Snell R Square	Nagelkerke R Square	
1	5408.871a	.001	.072	

a. Estimation terminated at iteration number 11 because parameter estimates changed by less than .001.	


Classification Tablea	
	Observed	Predicted	
		op_hernia	Percentage Correct	
		0	1		
Step 1	op_hernia	0	439654	0	100.0	
		1	359	0	.0	
	Overall Percentage			99.9	

a. The cut value is .500	


Variables in the Equation	
	B	S.E.	Wald	df	Sig.	Exp(B)	95% C.I.for EXP(B)	
							Lower	Upper	
Step 1a	Sectio	1.209	.113	114.475	1	.000	3.350	2.684	4.180	
	BVIKT_median_alla	.068	.108	.396	1	.529	1.070	.867	1.321	
	roker	.704	.165	18.268	1	.000	2.022	1.464	2.792	
	Morethan1_del	.779	.113	47.664	1	.000	2.179	1.747	2.718	
	BMI_3_gr			203.328	2	.000				
	BMI_3_gr(1)	.544	.139	15.302	1	.000	1.723	1.312	2.262	
	BMI_3_gr(2)	1.751	.125	195.827	1	.000	5.762	4.508	7.363	
	Ålder_3_gr			27.667	2	.000				
	Ålder_3_gr(1)	.304	.126	5.858	1	.016	1.355	1.060	1.733	
	Ålder_3_gr(2)	.742	.141	27.484	1	.000	2.099	1.591	2.770	
	pre_ecl_cat	-.047	.196	.058	1	.810	.954	.650	1.400	
	Constant	-8.743	.150	3406.593	1	.000	.000			

a. Variable(s) entered on step 1: Sectio, BVIKT_median_alla, roker, Morethan1_del, BMI_3_gr, Ålder_3_gr, pre_ecl_cat.	

Abdominal pain

Logistic Regression


Case Processing Summary	
Unweighted Casesa	N	Percent	
Selected Cases	Included in Analysis	440013	91.4	
	Missing Cases	41355	8.6	
	Total	481368	100.0	
Unselected Cases	0	.0	
Total	481368	100.0	

a. If weight is in effect, see classification table for the total number of cases.	


Dependent Variable Encoding	
Original Value	Internal Value	
0	0	
1	1	


Categorical Variables Codings	
	Frequency	Parameter coding	
		(1)	(2)	
Ålder_3_gr	1.00	259634	.000	.000	
	2.00	126164	1.000	.000	
	3.00	54215	.000	1.000	
BMI_3_gr	1.00	296686	.000	.000	
	2.00	98910	1.000	.000	
	3.00	44417	.000	1.000	


Block 0: Beginning Block


Classification Tablea,b	
	Observed	Predicted	
		ab_pain	Percentage Correct	
		0	1		
Step 0	ab_pain	0	358585	0	100.0	
		1	81428	0	.0	
	Overall Percentage			81.5	

a. Constant is included in the model.	
b. The cut value is .500	


Variables in the Equation	
	B	S.E.	Wald	df	Sig.	Exp(B)	
Step 0	Constant	-1.482	.004	145833.889	1	.000	.227	


Block 1: Method = Enter


Omnibus Tests of Model Coefficients	
	Chi-square	df	Sig.	
Step 1	Step	9581.346	9	.000	
	Block	9581.346	9	.000	
	Model	9581.346	9	.000	


Model Summary	
Step	-2 Log likelihood	Cox & Snell R Square	Nagelkerke R Square	
1	411931.204a	.022	.035	

a. Estimation terminated at iteration number 4 because parameter estimates changed by less than .001.	


Classification Tablea	
	Observed	Predicted	
		ab_pain	Percentage Correct	
		0	1		
Step 1	ab_pain	0	358513	72	100.0	
		1	81381	47	.1	
	Overall Percentage			81.5	

a. The cut value is .500	


Variables in the Equation	
	B	S.E.	Wald	df	Sig.	Exp(B)	95% C.I.for EXP(B)	
							Lower	
Step 1a	Sectio	.344	.011	1045.978	1	.000	1.411	1.382	
	BVIKT_median_alla	-.059	.008	55.826	1	.000	.943	.928	
	roker	.598	.014	1803.717	1	.000	1.819	1.770	
	Morethan1_del	.422	.008	2675.799	1	.000	1.525	1.501	
	BMI_3_gr			899.842	2	.000			
	BMI_3_gr(1)	.107	.010	124.747	1	.000	1.113	1.092	
	BMI_3_gr(2)	.369	.012	876.607	1	.000	1.446	1.411	
	Ålder_3_gr			2809.254	2	.000			
	Ålder_3_gr(1)	-.449	.009	2242.652	1	.000	.638	.627	
	Ålder_3_gr(2)	-.450	.014	1089.623	1	.000	.637	.621	
	pre_ecl_cat	.044	.018	5.921	1	.015	1.045	1.009	
	Constant	-1.694	.009	36408.081	1	.000	.184		

Variables in the Equation	
	95% C.I.for EXP(B)	
	Upper	
Step 1a	Sectio	1.440	
	BVIKT_median_alla	.957	
	roker	1.870	
	Morethan1_del	1.550	
	BMI_3_gr		
	BMI_3_gr(1)	1.134	
	BMI_3_gr(2)	1.482	
	Ålder_3_gr		
	Ålder_3_gr(1)	.650	
	Ålder_3_gr(2)	.655	
	pre_ecl_cat	1.082	
	Constant		

a. Variable(s) entered on step 1: Sectio, BVIKT_median_alla, roker, Morethan1_del, BMI_3_gr, Ålder_3_gr, pre_ecl_cat.	

All diagnosis


Logistic Regression

Case Processing Summary	
Unweighted Casesa	N	Percent	
Selected Cases	Included in Analysis	440013	91.4	
	Missing Cases	41355	8.6	
	Total	481368	100.0	
Unselected Cases	0	.0	
Total	481368	100.0	

a. If weight is in effect, see classification table for the total number of cases.	


Dependent Variable Encoding	
Original Value	Internal Value	
0	0	
1	1	


Categorical Variables Codings	
	Frequency	Parameter coding	
		(1)	(2)	
Ålder_3_gr	1.00	259634	.000	.000	
	2.00	126164	1.000	.000	
	3.00	54215	.000	1.000	
BMI_3_gr	1.00	296686	.000	.000	
	2.00	98910	1.000	.000	
	3.00	44417	.000	1.000	


Block 0: Beginning Block


Classification Tablea,b	
	Observed	Predicted	
		any_diagnos	Percentage Correct	
		0	1		
Step 0	any_diagnos	0	357204	0	100.0	
		1	82809	0	.0	
	Overall Percentage			81.2	

a. Constant is included in the model.	
b. The cut value is .500	


Variables in the Equation	
	B	S.E.	Wald	df	Sig.	Exp(B)	
Step 0	Constant	-1.462	.004	143643.709	1	.000	.232	


Block 1: Method = Enter


Omnibus Tests of Model Coefficients	
	Chi-square	df	Sig.	
Step 1	Step	9697.505	9	.000	
	Block	9697.505	9	.000	
	Model	9697.505	9	.000	


Model Summary	
Step	-2 Log likelihood	Cox & Snell R Square	Nagelkerke R Square	
1	415880.947a	.022	.035	

a. Estimation terminated at iteration number 4 because parameter estimates changed by less than .001.	


Classification Tablea	
	Observed	Predicted	
		any_diagnos	Percentage Correct	
		0	1		
Step 1	any_diagnos	0	357084	120	100.0	
		1	82703	106	.1	
	Overall Percentage			81.2	

a. The cut value is .500	


Variables in the Equation	
	B	S.E.	Wald	df	Sig.	Exp(B)	95% C.I.for EXP(B)	
							Lower	
Step 1a	Sectio	.367	.011	1215.720	1	.000	1.444	1.414	
	BVIKT_median_alla	-.053	.008	45.787	1	.000	.948	.934	
	roker	.597	.014	1808.964	1	.000	1.817	1.768	
	Morethan1_del	.432	.008	2838.291	1	.000	1.541	1.517	
	BMI_3_gr			894.750	2	.000			
	BMI_3_gr(1)	.098	.010	106.880	1	.000	1.103	1.083	
	BMI_3_gr(2)	.367	.012	878.927	1	.000	1.444	1.409	
	Ålder_3_gr			2686.858	2	.000			
	Ålder_3_gr(1)	-.437	.009	2161.664	1	.000	.646	.634	
	Ålder_3_gr(2)	-.433	.013	1027.925	1	.000	.649	.632	
	pre_ecl_cat	.043	.018	5.841	1	.016	1.044	1.008	
	Constant	-1.688	.009	36503.541	1	.000	.185		

Variables in the Equation	
	95% C.I.for EXP(B)	
	Upper	
Step 1a	Sectio	1.474	
	BVIKT_median_alla	.963	
	roker	1.868	
	Morethan1_del	1.566	
	BMI_3_gr		
	BMI_3_gr(1)	1.124	
	BMI_3_gr(2)	1.479	
	Ålder_3_gr		
	Ålder_3_gr(1)	.658	
	Ålder_3_gr(2)	.666	
	pre_ecl_cat	1.081	
	Constant		

a. Variable(s) entered on step 1: Sectio, BVIKT_median_alla, roker, Morethan1_del, BMI_3_gr, Ålder_3_gr, pre_ecl_cat.	

All except abdominal pain


Logistic Regression


Case Processing Summary	
Unweighted Casesa	N	Percent	
Selected Cases	Included in Analysis	440013	91.4	
	Missing Cases	41355	8.6	
	Total	481368	100.0	
Unselected Cases	0	.0	
Total	481368	100.0	

a. If weight is in effect, see classification table for the total number of cases.	


Dependent Variable Encoding	
Original Value	Internal Value	
.00	0	
1.00	1	


Categorical Variables Codings	
	Frequency	Parameter coding	
		(1)	(2)	
Ålder_3_gr	1.00	259634	.000	.000	
	2.00	126164	1.000	.000	
	3.00	54215	.000	1.000	
BMI_3_gr	1.00	296686	.000	.000	
	2.00	98910	1.000	.000	
	3.00	44417	.000	1.000	


Block 0: Beginning Block


Classification Tablea,b	
	Observed	Predicted	
		All_but_ab	Percentage Correct	
		.00	1.00		
Step 0	All_but_ab	.00	437117	0	100.0	
		1.00	2896	0	.0	
	Overall Percentage			99.3	

a. Constant is included in the model.	
b. The cut value is .500	


Variables in the Equation	
	B	S.E.	Wald	df	Sig.	Exp(B)	
Step 0	Constant	-5.017	.019	72409.661	1	.000	.007	


Block 1: Method = Enter


Omnibus Tests of Model Coefficients	
	Chi-square	df	Sig.	
Step 1	Step	1247.049	9	.000	
	Block	1247.049	9	.000	
	Model	1247.049	9	.000	


Model Summary	
Step	-2 Log likelihood	Cox & Snell R Square	Nagelkerke R Square	
1	33621.809a	.003	.037	

a. Estimation terminated at iteration number 8 because parameter estimates changed by less than .001.	


Classification Tablea	
	Observed	Predicted	
		All_but_ab	Percentage Correct	
		.00	1.00		
Step 1	All_but_ab	.00	437117	0	100.0	
		1.00	2896	0	.0	
	Overall Percentage			99.3	

a. The cut value is .500	


Variables in the Equation	
	B	S.E.	Wald	df	Sig.	Exp(B)	95% C.I.for EXP(B)	
							Lower	
Step 1a	Sectio	1.033	.042	615.305	1	.000	2.810	2.590	
	BVIKT_median_alla	.130	.038	11.780	1	.001	1.139	1.057	
	roker	.488	.064	57.473	1	.000	1.629	1.436	
	Morethan1_del	.760	.041	351.647	1	.000	2.139	1.976	
	BMI_3_gr			292.149	2	.000			
	BMI_3_gr(1)	-.041	.049	.700	1	.403	.960	.873	
	BMI_3_gr(2)	.785	.049	261.315	1	.000	2.191	1.993	
	Ålder_3_gr			24.536	2	.000			
	Ålder_3_gr(1)	.006	.044	.017	1	.897	1.006	.923	
	Ålder_3_gr(2)	.263	.055	22.723	1	.000	1.301	1.168	
	pre_ecl_cat	-.082	.079	1.077	1	.299	.921	.789	
	Constant	-5.974	.048	15193.402	1	.000	.003		

Variables in the Equation	
	95% C.I.for EXP(B)	
	Upper	
Step 1a	Sectio	3.049	
	BVIKT_median_alla	1.227	
	roker	1.848	
	Morethan1_del	2.316	
	BMI_3_gr		
	BMI_3_gr(1)	1.056	
	BMI_3_gr(2)	2.410	
	Ålder_3_gr		
	Ålder_3_gr(1)	1.096	
	Ålder_3_gr(2)	1.450	
	pre_ecl_cat	1.075	
	Constant		

a. Variable(s) entered on step 1: Sectio, BVIKT_median_alla, roker, Morethan1_del, BMI_3_gr, Ålder_3_gr, pre_ecl_cat.	

Uterine rupture


Logistic Regression


Case Processing Summary	
Unweighted Casesa	N	Percent	
Selected Cases	Included in Analysis	440013	91.4	
	Missing Cases	41355	8.6	
	Total	481368	100.0	
Unselected Cases	0	.0	
Total	481368	100.0	

a. If weight is in effect, see classification table for the total number of cases.	


Dependent Variable Encoding	
Original Value	Internal Value	
0	0	
1	1	


Categorical Variables Codings	
	Frequency	Parameter coding	
		(1)	(2)	
Ålder_3_gr	1.00	259634	.000	.000	
	2.00	126164	1.000	.000	
	3.00	54215	.000	1.000	
BMI_3_gr	1.00	296686	.000	.000	
	2.00	98910	1.000	.000	
	3.00	44417	.000	1.000	


Block 0: Beginning Block


Classification Tablea,b	
	Observed	Predicted	
		ut_rupture_tot	Percentage Correct	
		0	1		
Step 0	ut_rupture_tot	0	439440	0	100.0	
		1	573	0	.0	
	Overall Percentage			99.9	

a. Constant is included in the model.	
b. The cut value is .500	


Variables in the Equation	
	B	S.E.	Wald	df	Sig.	Exp(B)	
Step 0	Constant	-6.642	.042	25248.463	1	.000	.001	


Block 1: Method = Enter


Omnibus Tests of Model Coefficients	
	Chi-square	df	Sig.	
Step 1	Step	1923.032	9	.000	
	Block	1923.032	9	.000	
	Model	1923.032	9	.000	


Model Summary	
Step	-2 Log likelihood	Cox & Snell R Square	Nagelkerke R Square	
1	6835.871a	.004	.221	

a. Estimation terminated at iteration number 11 because parameter estimates changed by less than .001.	


Classification Tablea	
	Observed	Predicted	
		ut_rupture_tot	Percentage Correct	
		0	1		
Step 1	ut_rupture_tot	0	439440	0	100.0	
		1	573	0	.0	
	Overall Percentage			99.9	

a. The cut value is .500	


Variables in the Equation	
	B	S.E.	Wald	df	Sig.	Exp(B)	95% C.I.for EXP(B)	
							Lower	Upper	
Step 1a	Sectio	4.009	.133	906.026	1	.000	55.103	42.442	71.540	
	BVIKT_median_alla	.050	.086	.340	1	.560	1.051	.889	1.244	
	roker	-.028	.185	.023	1	.881	.973	.677	1.397	
	Morethan1_del	2.000	.113	312.624	1	.000	7.389	5.920	9.223	
	BMI_3_gr			.093	2	.954				
	BMI_3_gr(1)	-.021	.100	.043	1	.836	.980	.805	1.192	
	BMI_3_gr(2)	.021	.125	.027	1	.869	1.021	.799	1.304	
	Ålder_3_gr			6.381	2	.041				
	Ålder_3_gr(1)	.019	.093	.044	1	.834	1.020	.851	1.222	
	Ålder_3_gr(2)	-.300	.129	5.374	1	.020	.741	.575	.955	
	pre_ecl_cat	-.049	.141	.119	1	.730	.952	.723	1.256	
	Constant	-10.153	.172	3489.973	1	.000	.000			

a. Variable(s) entered on step 1: Sectio, BVIKT_median_alla, roker, Morethan1_del, BMI_3_gr, Ålder_3_gr, pre_ecl_cat.	

Placenta previa


Logistic Regression


Case Processing Summary	
Unweighted Casesa	N	Percent	
Selected Cases	Included in Analysis	440013	91.4	
	Missing Cases	41355	8.6	
	Total	481368	100.0	
Unselected Cases	0	.0	
Total	481368	100.0	

a. If weight is in effect, see classification table for the total number of cases.	


Dependent Variable Encoding	
Original Value	Internal Value	
0	0	
1	1	


Categorical Variables Codings	
	Frequency	Parameter coding	
		(1)	(2)	
Ålder_3_gr	1.00	259634	.000	.000	
	2.00	126164	1.000	.000	
	3.00	54215	.000	1.000	
BMI_3_gr	1.00	296686	.000	.000	
	2.00	98910	1.000	.000	
	3.00	44417	.000	1.000	


Block 0: Beginning Block


Classification Tablea,b	
	Observed	Predicted	
		pl_previa_tot	Percentage Correct	
		0	1		
Step 0	pl_previa_tot	0	438705	0	100.0	
		1	1308	0	.0	
	Overall Percentage			99.7	

a. Constant is included in the model.	
b. The cut value is .500	


Variables in the Equation	
	B	S.E.	Wald	df	Sig.	Exp(B)	
Step 0	Constant	-5.815	.028	44102.515	1	.000	.003	


Block 1: Method = Enter


Omnibus Tests of Model Coefficients	
	Chi-square	df	Sig.	
Step 1	Step	4338.107	9	.000	
	Block	4338.107	9	.000	
	Model	4338.107	9	.000	


Model Summary	
Step	-2 Log likelihood	Cox & Snell R Square	Nagelkerke R Square	
1	13494.687a	.010	.247	

a. Estimation terminated at iteration number 11 because parameter estimates changed by less than .001.	


Classification Tablea	
	Observed	Predicted	
		pl_previa_tot	Percentage Correct	
		0	1		
Step 1	pl_previa_tot	0	438705	0	100.0	
		1	1308	0	.0	
	Overall Percentage			99.7	

a. The cut value is .500	


Variables in the Equation	
	B	S.E.	Wald	df	Sig.	Exp(B)	95% C.I.for EXP(B)	
							Lower	Upper	
Step 1a	Sectio	4.215	.109	1489.856	1	.000	67.726	54.676	83.892	
	BVIKT_median_alla	-.952	.063	231.295	1	.000	.386	.342	.436	
	roker	-.447	.146	9.359	1	.002	.640	.480	.852	
	Morethan1_del	.306	.057	28.557	1	.000	1.358	1.214	1.519	
	BMI_3_gr			31.164	2	.000				
	BMI_3_gr(1)	-.276	.069	15.821	1	.000	.759	.662	.869	
	BMI_3_gr(2)	-.449	.098	20.836	1	.000	.638	.526	.774	
	Ålder_3_gr			128.349	2	.000				
	Ålder_3_gr(1)	.492	.070	49.399	1	.000	1.635	1.425	1.875	
	Ålder_3_gr(2)	.813	.072	127.399	1	.000	2.254	1.957	2.595	
	pre_ecl_cat	-.984	.131	56.570	1	.000	.374	.289	.483	
	Constant	-8.228	.117	4909.372	1	.000	.000			

a. Variable(s) entered on step 1: Sectio, BVIKT_median_alla, roker, Morethan1_del, BMI_3_gr, Ålder_3_gr, pre_ecl_cat.	
